# Effect of Vitamin D on Paraxonase-1, Total Antioxidant Capacity, and 8-Isoprostan in Children with Attention Deficit Hyperactivity Disorder

**DOI:** 10.1155/2022/4836731

**Published:** 2022-01-31

**Authors:** Niyaz Mohammadzadeh Honarvar, Mahsa Samadi, Marzieh Seyedi Chimeh, Fatemeh Gholami, Niki Bahrampour, Mahmoud Jalali, Mohammad Effatpanah, Mir Saeid Yekaninejad, Mina Abdolahi, Maryam Chamari

**Affiliations:** ^1^Department of Cellular and Molecular Nutrition, School of Nutritional Sciences and Dietetics, Tehran University of Medical Sciences, Tehran, Iran; ^2^Department of Nutrition, Science and Research Branch Islamic Azad University (SRBIAU), Tehran, Iran; ^3^School of Medicine, Ziaeian Hospital, International Campus, Tehran University of Medical Sciences, Tehran, Iran; ^4^Department of Epidemiology and Biostatistics, School of Public Health, Tehran University of Medical Science, Tehran, Iran

## Abstract

**Method:**

In this double-blind, randomized, placebo-controlled trial, 75 children (aged 6–12) diagnosed with ADHD were randomly assigned into two groups. The supplementation group received vitamin D3 (2000 IU), and the control group received a placebo for 3 months. Blood samples were collected at baseline and after intervention to analyze the 25(OH)D, paraxonase-1 activity (PON-1), Total Antioxidant Capacity (TAC), and 8-isoprostan levels.

**Results:**

A significant rise in circulating 25(OH)D was observed in the vitamin D group versus the placebo group at the end of the study. There was no reduction in 8-isoprostan levels in the vitamin D group compared to the placebo group. Serum paraxonase-1 and TAC concentration decreased in both groups, but these alterations were not statistically significant in the treatment group versus the placebo group at the end of the intervention.

**Conclusion:**

Vitamin D supplementation for 3 months did not have beneficial effects on biomarkers of oxidative stress status. To confirm these findings, further studies on children are suggested.

## 1. Introduction

Attention deficit hyperactivity disorder (ADHD) is one of the most prevalent neuropsychiatric disorders in childhood [1]. The prevalence of ADHD in children and adolescents is about 6-7%, and it is more frequent in boys than girls [2]. The main symptoms of ADHD are inability to concentrate and pay attention, hyperactivity, impulsivity, and difficulties in learning behavior and psychosocial adjustment, which may persist into adulthood [3–5]. The exact etiology of ADHD is unknown, but there are various hypotheses [6]. One of these is that oxidative stress is involved in various pathologies underlying many disorders, such as bipolar disorder, schizophrenia, and depression [7, 8]. Oxidative stress is emphasized as an important mechanism in the destruction, damage, and death of cells. Recent studies have suggested the important role of oxidative stress in the pathogenesis of many mental disorders [9]. Several studies have investigated the oxidative stress status in children and adolescents with ADHD. Some of these studies have found that there is an imbalance between oxidant (reactive oxygen species (ROS)) and antioxidant defense in ADHD patients and concluded that oxidative stress was higher in people with ADHD [10–17]. ROS is known to destroy polyunsaturated fatty acids (PUFAs) and Deoxyribonucleic Acid (DNA) of brain neurons through oxidation. Oxidative stress and neuroinflammation can activate astrocytes and microglia and cause secretion of proinflammatory cytokines and catecholaminergic dysregulation. These mechanisms can increase the symptoms of ADHD [18].

Vitamin D is not only involved in bone metabolism and serum calcium regulation but also has significant effects on brain function. There is evidence demonstrating the widespread presence of vitamin D receptors and 1*α*-hydroxylase (the enzyme responsible for the formation of the active vitamin) in the human brain [19], as well as possibly playing a role in the etiopathogenesis of psychiatric diseases such as autism, schizophrenia, depression, and ADHD [20–23]. In the early stages of life, vitamin D deficiency might harm neuronal development and function [20]. Observationally, there are limited studies focused on the association between vitamin D and hyperactivity and behavioral problems in children. Some of these studies have indicated that serum vitamin D level was significantly lower in children and adolescents with ADHD, compared to healthy controls [24–27]. Recent systematic review and meta-analyses showed that children and adolescents with ADHD have lower mean concentration of serum 25(OH)D than healthy children [28]. The results of some experimental studies have reported that vitamin D may have antioxidant properties by modulating some antioxidant enzyme activities [29–31]. It has also been considered as a membrane antioxidant [32], and additionally, its antioxidant potential was considered to be equal or greater than that of vitamin E and melatonin [33, 34]. Pharmaceutical interventions such as Ritalin are effective in reducing the symptoms of ADHD; however, there is significant interest in other evidence-based treatment procedures [35]. Therefore, the aim of this study was to investigate the effect of vitamin D supplementation on oxidative stress indices, including paraxonase-1 activity, total antioxidant capacity, and 8-isoprostan in children with ADHD.

## 2. Methods

### 2.1. Trial Design and Setting

This study was a randomized, double-blind, placebo-controlled clinical trial conducted on 86 children aged 6–12. Subjects were referred from Ziaeian Hospital (Tehran, Iran) to participate in the study, from December 2015 to September 2016. The ethnicities of Iranian participants were mostly Persian, Tork, Gilak, Mazani, Kord, and Lor [36], and the color of their skin was yellowish. The study was approved by the Human Experimentation Ethics Committee of Tehran University of Medical Sciences (IR.TUMS.REC.1394.1719) and was registered in the Iranian Registry of Clinical Trials under the registry number IRCT2016102324081N2. At the beginning of the study, the children's parents were given an oral and written explanation of the study, including its benefits and procedures, and were asked to read and sign an informed consent document.

### 2.2. Trial Participants

Children aged 6–12 years with ADHD were selected according to the criteria of the Diagnostic and Statistical Manual of Mental Disorders, Fifth Edition (DSM-V). All the participants received Ritalin. Children diagnosed less than one year with BMI <95th percentile were included in the study. Also, the duration of consumption of Ritalin should be less than one year too. Exclusion criteria were any disease (infectious or cardiovascular diseases, diabetes, hypertension, hyperthyroidism, digestive diseases, liver or kidney diseases, respiratory diseases and allergies, and neurological diseases); a history of severe head trauma; BMI >95th percentile; and taking any supplement such as omega3, calcium, vitamin D, and any medicine except Ritalin.

### 2.3. Intervention

Participants were randomly assigned to receive either vitamin D3 or a placebo. This trial was originally designed to examine the therapeutic effects of vitamin D3 in patients with ADHD. The patients were randomly allocated into two study groups using a permuted-block randomization method: the treatment and placebo group. Blood samples were obtained at the baseline and 3 months after intervention. Patients were considered compliant if they consumed more than 80% of the provided medication. Compliance with the consumption of vitamin D supplements and placebos was monitored once a week by phone interviews. The dietary intakes of participants were assessed by a validated 24-h recall covering 3 days (including one weekend day and two weekdays) at the beginning and at the end of the intervention period [37]. They were asked to explain what they ate by a trained dietitian.

### 2.4. Assessment of Variables

Anthropometric indices, including weight, height, and BMI, were obtained through comprehensive interviews and physical examinations. Body weight was measured to the nearest 0.1 kg using a Seca scale, with subjects minimally clothed, without shoes, and in an overnight fasting state. Birth weight of the subjects was obtained from their medical records. Height was measured to the nearest 0.1 cm using a nonstretched tape measure (Seca), without shoes. Lastly, sex-specific BMI percentiles were calculated using the SAS program for the 2000 CDC Growth Charts for the United States [38]. BMI < 5 (underweight), BMI = 5–85 (healthy), and BMI = 85–95 (overweight) were classified. Sun exposure of each participant was assessed via the validated questionnaire. The questionnaire scored the amount of time spent outdoors each day and the amount of skin exposed [39].

### 2.5. Sample Size Determination

Sample size in this study was estimated for the expected change of three biomarkers (TAC, Pox1, and 8-isoprostan) with support of the literature [40–42]; also, standard deviation as a dispersion parameter was obtained from those studies [40–42], with a power of 80% (1 − *β* = 0.8) and a type I error *α* = 0.05 and 10% attrition rate, and a final sample size of 80 (40 patients in each group) was estimated.

### 2.6. Randomization and Drug Allocation

Permuted-block randomization was used to allocate randomized individuals to the two groups, so there was no difference in terms of gender and age. A statistician was responsible for the generation of randomization codes. Sequentially numbered, sealed, and stapled packages were used to conceal allocation. A third party was responsible for the random allocation and rating of patients. The participants, the physician who prescribed the medications, and the nutritionist were blinded to the allocated treatment. According to previous studies, 2000 IU of vitamin D is an optimum dosage for preventing diseases in children and adults without any side effects [43, 44]. The intervention group received two tablets of vitamin D3 (1000 IU vitamin D3 per each tablet) daily for 3 months. The second group received identical-looking placebo tablets. All vitamin D tablets and their placebos were the same color and shape and were produced by Jalinous Company (Tehran, Iran). All the participants received their oral Ritalin as prescribed by their neurologist. The participants were asked not to take any vitamins or supplements during the trial.

### 2.7. Laboratory Methods

At the beginning and the end of the 3 months' supplementation trial, 10 cc of fasting blood samples was collected from the patients, and the serum was isolated and frozen at −80°C. Serum 25-hydroxyvitamin D [25(OH)D] was assayed using a commercial ELISA kit (25-Hydroxyvitamin D ELISA kit; DIAsource ImmunoAssays S.A.). Serum concentrations of PON-1, TAC, and 8-isoprostan were determined by enzyme-linked immunosorbent assay (ELISA kit; Bioassay Technology Laboratory, China).

### 2.8. Statistical Analysis

Data processing and analysis were performed using SPSS for Windows (SPSS, version 20; Chicago, IL, USA). Normally distributed data were expressed as mean (±standard error). Baseline characteristics and dietary intakes of the treatment and placebo groups were compared using an independent-sample *t*-test. To determine the effects of vitamin D supplementation on biomarkers of oxidative stress and serum 25(OH) vitamin D level, univariate covariance analysis (ANCOVA) adjusted for the baseline vitamin D was used. Categorical variables were compared using the chi-square test. The significance level was set at *P* < 0.05.

## 3. Results

### 3.1. Baseline Characteristics of Study Participants

A total of 86 children with ADHD were chosen in our study. Five patients among the children in the vitamin D group were excluded during the trial because of the reasons listed: changing their physician (*n* = 2), no tendency to consumption of Ritalin (*n* = 2), and catching cold and stopping drug consumption (*n* = 1). In the placebo group, 6 patients were excluded because of no tendency to consumption of Ritalin. So, a total of 75 participants (vitamin D (*n* = 37) and placebo (*n* = 38)) completed the trial. The baseline characteristics of the participants are shown in Table 1. The distributions of age, gender, actual weight, and birth weight did not differ significantly between the treatment and placebo groups.

Obesity was determined by the BMI percentile in the two groups. There was no significant difference in the BMI percentile between the placebo and control group (Table 2).

### 3.2. Dietary Intake

Dietary intakes of energy, carbohydrates, fat, protein, vitamin D, vitamin C, vitamin E, selenium, calcium, EPA, DHA, and beta-caroten did not differ between the groups, *P* > 0.05 (Table 3).

### 3.3. Circulating 25(OH)D

A significant rise in circulating 25(OH)D was observed in the vitamin D group versus the placebo group by the end of the study (*P* value = 0.01) (Table 4).

### 3.4. Sun Exposure

The mean of daily sunlight exposure scores of the vitamin D group and placebo group was 12.43 ± 1.38 and 13.58 ± 1.35, respectively, and there were not any statistically differences between them [45].

### 3.5. Oxidative Stress Biomarkers

At the beginning of the study, there was no significant difference in the variables of oxidative stress between the placebo and control group, as shown in Table 5. However, there was a significant difference between the two groups at the vitamin D baseline.

The results of univariate covariance analysis showed that, after adjusting for vitamin D at baseline, there was no significant reduction in 8-isoprostan as an oxidative stress marker in both the treatment and placebo groups (*P* < 0.05) after 3 months. Also, at the end of the study, there was no significant difference in paraxonase-1 and serum TAC levels in the vitamin D group versus the placebo group.

## 4. Discussion

In the present study, it was observed that vitamin D supplementation for 3 months resulted in a significant increase in 25(OH)D concentration in children with ADHD. As an improvement in vitamin D status, there was no significant increase in concentrations of plasma PON-1 and TAC and no significant decrease in 8-isoprostan as a biomarker of oxidative stress in the vitamin D group, compared with the placebo. To the best of the authors' knowledge, this clinical trial is the first that assesses the effect of vitamin D supplementation on these oxidative stress biomarkers (PON-1, TAC, and 8-isoprostan) in children with ADHD. It is necessary to mention that all children in both groups were taking methyl phenidate throughout the study.

The antioxidative action of vitamin D was discovered first to protect ROS-induced neurotoxicity by regulating proteins which reduce oxidative stress [46]. In 1993, Wiseman showed that vitamin D may accumulate in the membrane to prevent lipid peroxidation. In addition, reducing membrane fluidity also inhibits lipid peroxidation [32]. In another study, it was shown that vitamin D has equal or greater antioxidant properties than vitamin E [33]. Several interventional studies have indicated the effect of vitamin D supplementation on oxidative stress biomarkers and have produced conflicting results.

Findings from the current study revealed that vitamin D supplementation had no significant effect on 8-isoprostan and PON-1 concentrations, compared with placebo-taking children with ADHD. This finding is in agreement with a previous study, in which investigators did not find a significant effect of vitamin D on serum malondialdehyde (MDA) concentrations. Tabasi et al. showed that, in 60 patients with endometrial hyperplasia, vitamin D supplementation (50,000 IU twice a week for 12 weeks) improved total antioxidant capacity during intervention, but showed no significant changes in MDA levels [47]. Cavalcante et al. showed that high-dose vitamin D supplementation (400,000 IU for 4 weeks) in elderly women with vitamin D deficiency led to significant increases in total antioxidant capacity; however, the MDA level increased in both the control and the intervention groups [48]. In contrast, others showed a significant reduction of serum MDA. In another study, supplementation with 50,000 IU every 2 weeks versus placebo for 4 months in nonalcoholic fatty liver disease (NAFLD) patients lowered serum MDA, but there were no significant changes in total antioxidant levels in the vitamin D group [49]. Grunwald et al. observed that improvements in 25OHD levels led to reductions in urinary 8-isoprostane in seven patients with vitamin D deficiency/insufficiency [50].

This study demonstrated that vitamin D supplementation did not increase TAC in both groups. In contrast to these findings, Sepehrmanesh et al. showed significant changes in the serum levels of total antioxidant capacity and glutathione in patients with major depressive disorder [51]. Similar findings were also observed in Asemi et al.'s study, in their clinical, randomized vitamin D supplementation trial (400 IU daily) among healthy pregnant women, which resulted in a significant increase in total antioxidant capacity and glutathione in a vitamin D group versus a placebo over 9 weeks [52].

Although the exact mechanisms of the effects of vitamin D on oxidative stress biomarkers are not known [52], multiple cellular and molecular mechanisms have been suggested to explain this association. Multiple mechanisms might be involved in vitamin D-mediated reduction of oxidative stress biomarkers: (1) vitamin D can inhibit iNOS synthesis, so nitric oxide production decreases in the brain (which is a cause of neuronal damage); (2) vitamin D can stimulate the expression and activity of gamma-glutamyl transpeptidase (GGT), which participates in the glutathione cycle between neurons and astrocytes; and (3) vitamin D increases glutathione levels to protect neurons, so it can lead to decreased ROS [46, 53, 54]. Vitamin D is effective in increasing GSH levels by about 50% [33].

In this study, a significant change in TAC, paraxonase-1, and 8-isoprostan was not observed in the vitamin D group compared to the placebo group. Although oxidative stress plays a role in the pathophysiology of many chronic conditions, limited information is available on the effect of vitamin D supplementation on oxidative stress. No clinical trial has been carried out which assesses the effect of this vitamin in children; maybe the effect of vitamin D on oxidative stress biomarkers in children differs from adults, and further studies are needed. On the other hand, some animal studies have shown that methylphenidate can lead to increased oxidative stress; therefore, a trial without the use of methylphenidate could be more effective in evaluating the effect of vitamin D on oxidative stress [55, 56].

Currently, no official agreement has been reached regarding blood levels of 25(OH)D that indicate deficiency, insufficiency, and sufficiency of vitamin D, especially related to brain health. For example, serum levels of 25(OH)D may be low despite a person having an adequate diet or sufficient sun exposure, due to its conversion to other forms such as 1,25(OH)D. Many practitioners favor 25(OH)D serum levels of at least 30 ng/ml or 75 nmol/L, although for treatment of cancer, diabetes, autoimmune illness, or depression, levels of 60 to 80 ng/ml may be beneficial [57]. Also, vitamin D is widely accepted to have an anti-inflammatory role in human pathophysiology [58, 59], but based on a systematic review by Calton et al., no evidence of a benefit of cholecalciferol supplementation on systemic inflammatory markers was observed, and 25(OH)D levels above 80 nmol/L could be considered effective in decreasing inflammatory markers [58]. In the current clinical trial, the 25(OH)D serum mean level increased to 33.44 nmol/L, and perhaps a serum level over 50 nmol/L could be considered as the target level to observe the antioxidant effects of vitamin D. Increasing supplementation can help improve 25(OH) vitamin D levels.

## 5. Limitation

The current study has some limitations. In summary, children used methylphenidate along with vitamin D, and it may increase oxidative stress. Also, another limitation is the short duration of supplementation. In other words, vitamin D3 supplementation for 3 months is not enough to elevate serum 25(OH) D to target levels and to influence the antioxidant system. Therefore, long-term vitamin D supplementation without methylphenidate is essential. In addition, it is obvious that serum levels of vitamin D do not reflect accurately the vitamin D status, so PTH measurement is suggested to evaluate its suppression [60]. Finally, we did not consider seasonality and UV exposure in this study.

## 6. Conclusions

Overall, the results of this clinical trial in children with ADHD show that vitamin D supplementation for 3 months did not have beneficial effects on the biomarkers of oxidative stress status. To confirm this finding, further studies on children are suggested.

## Figures and Tables

**Table 1:** Comparison of the baseline characteristics between the treatment and placebo group.

Variable	Vitamin D (*n* = 37)	Placebo control (*n* = 38)	*P* value
^*∗*^Gender, *n* (%)			0.24
Male	28 (75.7)	24 (63.2)	
Female	9 (24.3)	14 (36.8)	
Age (month)	100.65 (3.55)	106.18 (4.03)	0.30
Actual height (cm)	135.81 (2.26)	137.10 (1.81)	0.65
Actual weight (kg)	32.15 (2.13)	32.60 (1.69)	0.86
Birth weight (gr)	3237.30 (92.76)	3115.79 (110.34)	0.38
Sun exposure (daily score)	12.43 (1.38)	13.58 (1.35)	0.55

Independent-sample *t*-test; mean ± SD; SD: atandard error. ^*∗*^Chi-square; number (percent).

**Table 2:** Comparison of the BMI between treatment and placebo groups at the beginning of the study.

Variable		Placebo control	Vitamin D	*P* value
BMI (kg/m^2^)	<5	8 (21.1)	8 (21.6)	0.98
	5–85	19 (50.0)	19 (51.4)	
	85–95	11 (28.9)	10 (27.0)	
	95<	0	0	

Chi-square test; number (percent).

**Table 3:** Comparison of dietary intake between treatment and placebo groups at the beginning of the study.

Variable per day (%)	Placebo control	Vitamin D	*P* value
Energy (kcal)	1822.84 (32.83)	1835.13 (61.35)	0.85
CHO (g)	231.42 (6.35)	276.10 (36.01)	0.22
PRO (g)	55.11 (2.14)	58.77 (2.699)	0.29
FAT (g)	78.32 (2.31)	78.57 (2.79)	0.94
Vitamin E (mg/L)	23.05 (0.96)	23.82 (1.22)	0.62
Vitamin D (*μ*g)	2.69 (2.14)	2.95 (1.85)	0.92
Vitamin C (mg)	74.42 (12.02)	67.29 (7.78)	0.62
Selenium (*μ*g)	0.03 (0.004)	0.07 (0.03)	0.30
B-caroten (*μ*g)	697.30 (113.44)	798.46 (131.13)	0.56
EPA (g)	0.01 (0.005)	0.01 (0.005)	0.96
DHA (g)	0.03 (0.01)	0.57 (0.54)	0.31
Calcium (mg)	552.66 (34.76)	510.89 (69.16)	0.58

^*∗*^Independent-sample *t*-test; mean ± SD; SD: standard error.

**Table 4:** Comparison of the mean difference of variables at the beginning and end of the study (end-beginning) between the two groups.

Variable (%)	Placebo control		Vitamin D		*P* value
	Before	After	Before	After	
TAC (ng/ml)	18.57 (2.12)	17.69 (2.40)	19.22 (2.54)	14.90 (1.90)	0.07
PON-1 (pg/ml)	101.91 (12.83)	101.80 (13.31)	96.05 (13.37)	94.65 (12.69)	0.70
8-Isoprostane (pg/ml)	218.27 (34.73)	282.21 (49.98)	194.24 (32.86)	239.70 (41.50)	0.75
Vitamin D3 (ng/ml)	15.99 (1.88)	15.99 (1.88)	23.52 (1.75)	33.44 (2.14)	0.01

Univariate covariance analysis (ANCOVA) adjusted for the baseline vitamin D; ^*∗∗*^mean ± standard error; TAC: total antioxidant capacity; PON-1: paraxonase-1; 8-isoprostane; vitamin D3.

**Table 5:** Comparison of the baseline variable between the treatment and placebo group.

Variable (%)	Placebo control	Vitamin D	*P* value
TAC (ng/ml)	18.57 (2.12)	19.22 (2.54)	0.84
PON-1 (pg/ml)	101.91 (12.83)	96.05 (13.37)	0.75
8-Isoprostane (pg/ml)	218.27 (34.73)	194.24 (32.86)	0.61
Vitamin D (ng/ml)	15.99 (1.88)	23.52 (1.75)	0.05

Independent *t*-test; ^*∗∗*^mean ± standard error; TAC: total antioxidant capacity; PON-1: paraxonase-1; 8-isoprostane.

## Data Availability

Availability of data and materials will be granted if required.
